# Successful surgical sperm retrieval from a patient with 45,X/46,XY mosaicism followed by in vitro fertilization pregnancy: A case report

**DOI:** 10.1097/MD.0000000000022223

**Published:** 2020-10-02

**Authors:** Yu Zhang, Zheng Liu, Jian Ou, Ling-Xiao Zhang, Wei-Ying Lu, Qi Li, Yanlin Ma

**Affiliations:** a Hainan Provincial Key Laboratory for Human Reproductive Medicine and Genetic Research; b Hainan Provincial Clinical Research Center for Thalassemia, The First Affiliated Hospital of Hainan Medical University; c Key Laboratory of Tropical Translational Medicine of Ministry of Education, Hainan Medical University, Haikou, Hainan; d College of Medical Laboratory Science, Guilin Medical University, Guilin, Guangxi; e Center of Reproduction and Genetics, Affiliated Suzhou Hospital of Nanjing Medical University, Suzhou, Jiangsu, China.

**Keywords:** 45,X/46,XY mosaicism, in vitro fertilization, mixed gonadal dysgenesis

## Abstract

**Rationale:**

Mixed gonadal dysgenesis is a rare disorder of sex development, and typically contains a mosaic 45,X/46,XY karyotype.

**Patient concerns:**

We reported here a case of a 42-year-old man with infertility for 6 years and inability to ejaculate during intercourse.

**Diagnosis:**

Physical examination confirmed that the external genitalia was male. The right testis of this patient was resected and the left testis had intrascrotal calcification. Hormone test showed that the level of follicle-stimulating hormone was 20.14 IU/L (normal range, 1.27–19.26 IU/L). No deletion or mutation was found on the sex-determining region Y. H&E staining revealed seminiferous tubule dysgenesis. The karyotyping in peripheral blood and testicular tissue was 45,X/46,XY and 45,X/47,XYY/46,XY, respectively. Based on these results, the patient was diagnosed with 45,X/46,XY or 45,X/47,XYY/46,XY mosaicism and gonadal dysgenesis.

**Interventions:**

In vitro fertilization and embryo transfer technology were used to help his wife to achieve pregnancy.

**Outcomes:**

A normal baby boy was born at 36 weeks of gestation with a karyotype 46, XY.

**Lessons:**

We reported a rare case of a karyotype 45,X/46,XY in blood cells and 45,X/47, XYY/46,XY in testicular tissue. In vitro fertilization and embryo transfer technology can help to achieve pregnancy.

## 1 Introduction

Mixed gonadal dysgenesis (MGD) is a rare disorder of sex development associated with sex chromosome aneuploidy and mosaicism of the Y chromosome.^[1]^ At present, the etiology and pathogenesis of this disorder remain obscure, but sex chromosome abnormalities, abnormal gonadal development, and related endocrine disorders during embryonic development may be involved. Patients with 45,X/46,XY mosaicism is rare with an incidence of approximately 1.7/10,000 in newborns.^[2]^ The phenotypes of 45,X/46,XY mosaicism are highly heterogeneous, from women with Turner syndrome, men with ambiguous genitals to normal phenotypes.^[3]^ In the present study, we report a case of a male with a karyotype of 45X/46,XY in peripheral blood, but the karyotype in his testis is 45,X/47,XYY/46,XY. After careful assessment, testicular sperm aspiration was used to obtain his sperm, and in vitro fertilization and embryo transfer (IVF-ET) technology was used to achieve pregnancy for his wife.

## 2 Case presentation

### 2.1 Ethics

All procedures performed in this study were in accordance with the ethical standards defined by the Hainan Medical University Committee and the Helsinki declaration of 1975, as revised in 2008. Written informed consent for publication of this case report was obtained from the patient.

### 2.2 Clinical history

**Figure 1:**
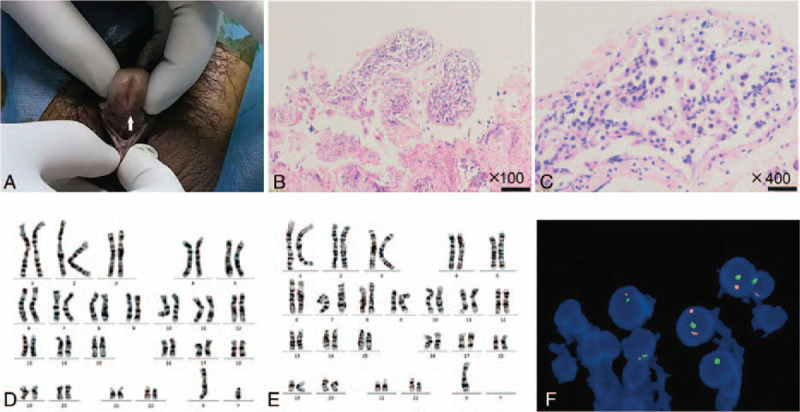
Diagnosis of 45,X/46,XY mosaicism. The patient's penis is short. The urethral opening (arrow) is on the middle and anterior parts of the ventral side of the penis (A). H&E staining at 100× (B) and 400× (C) showed seminiferous tubule dysgenesis. Chromosomal analysis showed a 46,XY karyotype in (D) and 45,X in (E). FISH analysis revealed a 45,X/46,XY/ 47,XYY karyotype in testicular tissue (F).

A 42-year-old man (height, 153 cm; weight, 57 kg) was presented to our hospital for inability to ejaculate during intercourse and infertility for 6 years. Physical examination revealed that the pubic hair exhibited male distribution and that the external genitalia was male. Hypospadias was present with the urethra opening located in the middle and anterior parts of the ventral side of the penis (Fig. 1A). His right testis was resected several years ago because of cryptorchidism. B-ultrasound of scrotum showed that the size of the left testis was 35 × 29 × 17 mm with the presence of intrascrotal calcification.

### 2.3 Hormone test

Following admission, blood tests, coagulation function tests, liver and renal function tests, and tests for infectious diseases were all normal. The level of follicle-stimulating hormone was 20.14 IU/L (normal range, 1.27–19.26 IU/L). Luteinizing hormone was 5.78 IU/L (normal range, 1.24–8.62 IU/L). Estradiol (E_2_) was 127 pmol/L (normal range, 73.4–172.5 pmol/L). Prolactin was 157.73 mIU/L (normal range, 56.0–278.3 mIU/L). Testosterone was 10.28 nmol/L (normal range, 6.07–27.1 nmol/L).

### 2.4 Azoospermia factor test

Eighteen loci microdeletions (SY81, SY182, SY86, SY121, SY124, SY127, SY128, SY133, SY134, SY130, SY145, SY153, SY242, SY239, SY152, SY254, SY255, SY157) of the Y chromosome azoospermia factor locus region were detected by multiple PCR. The results showed that there was no deletion or mutation on sex-determining region Y.

### 2.5 H&E staining, chromosomal analysis, and fluorescence in situ hybridization

Seminiferous tubule was obtained through the testicular sperm aspiration procedure and dysgenesis with decreased spermatogenesis was observed by H&E staining (Fig. 1B and C). Chromosomal analysis was performed and the result showed that there was a 45,X/46,XY karyotype in peripheral blood (Fig. 1D and E). To ensure a better characterization of abnormalities, fluorescence in situ hybridization was carried out by CEP X (DXZ1) SpectrumGreen/CEP Y (DYZ3) SpectrumOrange Probe Direct Labeled Fluorescent DNA Probe Kit (Vysis, Abbott Molecular). The results were (DXZ1 × 1) [205]/(DXZ1 × 1, DYZ3 × 2) [30]/(DXZ1 × 1, DYZ3 × 1) [775] after 1000 cells were calculated and analyzed. The karyotype of testicular tissue was determined as 45,X[205]/47,XYY [30]/46,XY[775] (Fig. 1F). However, karyotyping in peripheral blood showed 45,X [54]/46,XY [8] mosaicism.

### 2.6 Diagnosis and treatment

**Figure 2:**
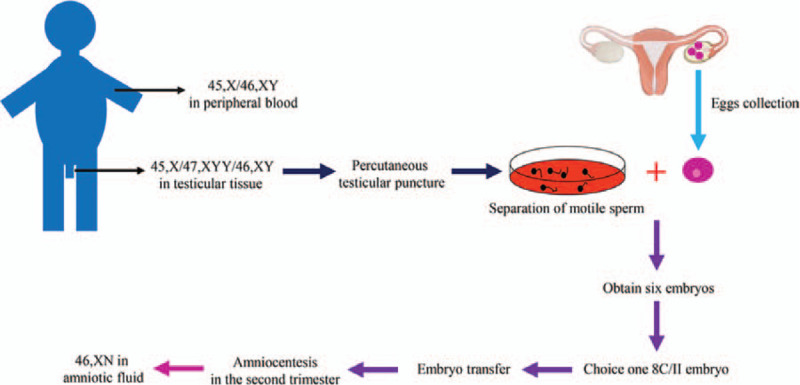
Workflow of in vitro fertilization and embryo transfer.

Based on the results of examinations and tests, the patient was diagnosed with 45,X/46,XY or 45,X/47,XYY/46,XY mosaicism with a diagnosis of gonadal dysgenesis. The patient declined to undergo a preimplantation genetic testing for aneuploidy (PGT-A). With the requirement of the patient, IVF-ET technology was chosen to help his wife to achieve pregnancy. Following testicular sperm aspiration, 6 viable embryos were obtained by intracytoplasmic sperm injection, and one 8C/II embryo was transferred 3 days later. During the second trimester, amniocentesis was performed and the karyotyping showed 46, XN. A normal baby boy was born at 36 weeks of gestation (Fig. 2).

## 3 Discussion

In 1963, Sohval^[4]^ first described MGD. Most of MGD patients had unilateral nonpalpable testis, contralateral streak gonad and persistent Mullerian structures.^[5]^ The mechanisms leading to MGD include incomplete inhibition of Mullerian tube development, incomplete differentiation of mesonephric tubules and hypoplasia of external genitalia.^[6]^ Robboy et al^[7]^ reported that Mullerian tissue was present in 95% of MGD patients. According to previous studies, the MGD patients have variable clinical manifestations. Chang et al^[2]^ reported that 95% of fetuses with 45,X/46,XY mosaicism had normal male external genitalia and Telvi et al^[8]^ reported that 60% of MGD patients had vulvar gender ambiguity, whereas the proportion of male external genitalia phenotype (bilateral testicles descending to scrotum) was the smallest (about 11%–12%). Rosa et al. reported that 29% of MGD patients had hypospadias and cryptorchidism.^[3]^ In addition, male MGD patients also often have short stature, which may be related to hypothalamic gonadal axis and gonadal dysfunction.^[9]^ Compared with the 45,X/46,XY male patients, patients with female external genitalia are more likely to have phenotypes of Turner syndrome, such as short neck or webbed neck, low hairline, shortening of the fourth metacarpal bone, and cardiovascular and renal abnormalities.^[10]^

In our case, the testis presented 3 nuclear chimeras, which were inconsistent with the karyotyping of peripheral blood. After searching in PubMed with keywords 45,X/46,XY/47,XYY and testicular tissues, there were no previous reports of the karyotype 45,X/46,XY/47,XYY in testicular tissues. In 1974, Osztovics et al^[11]^ first reported a phenotypic female patient with 45,X/46,XY/47,XYY mosaicism in lymphocytes. Khan et al^[12]^ previously reported a case of 45,X/45,Y/46,XY/46,YY/47,XYY in blood cells, which existed in different proportions. A number of studies have shown that the proportion of cells with 45,X/46,XY mosaicism in peripheral blood was not related to the phenotype, and that even significant phenotypic differences can be observed in identical twins.^[13]^ Akinsal et al^[14]^ found that in male patients without hypospadias and cryptorchidism, the proportion of 45,X cells could reach up to 73%. Nomura et al^[15]^ reported that the proportion of peripheral blood with 46,XY cells in the phenotypic male patients was lower than 0.2%, while that was 50% in gonads. Wu et al^[16]^ found that the proportion of 46,XY cells in phenotypic female patient could reach 60%. In our case, the proportion of 46,XY cells in peripheral blood was only 10%, whereas the proportion of XY cells in testis was 78%. Our findings can be explained by the facts that our patient had normal androgen level and testicular spermatogenic function because 46,XY cells in testis had a higher proportion.

In our case, the proportion of 45,X cells in gonad was lower than that in peripheral blood. The possible reason was that the 45,X cell arrested before meiosis. A study in mice showed that XO pachytene oocytes in the stage of meiosis could not be eliminated progressively due to the inability of X-chromosome synapsis, resulting in the inability to pass cell cycle checkpoints.^[17]^ Ren et al^[18]^ found that 12.4% of the 46,XY cells in 45,X/46,XY mosaicism had sex chromosome asynapsis, resulting in reduced sperm production. The reduced sperm production was also confirmed in 47, XYY, 47,XXY, and 46,XY/47,XXY chimeras.^[19]^ In our patient, a lower proportion (3%) of 47,XYY cells was found in the testis.

The limitation of our case report was the patient declined to undergo a PGT-A. PGT-A was designed to minimize chances of genetically abnormal embryos, and avoid miscarriage and live born trisomic offspring and to improve IVF success.^[20]^ Therefore, this test will benefit both IVF patients and prospective parents at risk of aneuploid offspring. Furthermore, the ability of PGT-A to detect mosaicism is limited by the number and location of trophectoderm cells in the biopsy.^[21]^ Although aneuploid mosaicism may be present in trophectoderm, the possibility of detecting mosaicism within a single trophectoderm biopsy represents a contemporary challenge to bring about improvement to the practice of PGT-A.

## 4 Conclusion

We report a rare case of a patient with 45,X/46,XY mosaicism in blood cells and 45,X/46,XY/47,XYY mosaicism in testicular tissues. By using IVF-ET technology, the patient’ wife achieved pregnancy. To the best of our knowledge, this is first report of using IVF-ET technology to achieve pregnancy for the wife of a male patient with 45,X/46,XY/47,XYY mosaicism.

## Author contributions

**Conceptualization:** Yu Zhang, Yanlin Ma.

**Funding acquisition:** Qi Li, Yanlin Ma.

**Investigation:** Jian Ou, Ling-Xiao Zhang, Wei-Ying Lu.

**Methodology:** Yu Zhang, Zheng Liu, Qi Li, Yanlin Ma.

**Writing – original draft:** Yu Zhang, Zheng Liu.

**Writing – review & editing:** Yu Zhang, Zheng Liu, Yanlin Ma.
